# Pharmacology and toxicology of the novel investigational agent Cantrixil (TRX-E-002-1)

**DOI:** 10.1007/s00280-016-3224-2

**Published:** 2016-12-24

**Authors:** Muhammad Wasif Saif, Andrew Heaton, Kimberley Lilischkis, James Garner, David M. Brown

**Affiliations:** 1 Department of Medicine and Cancer Center, Tufts Medical Center, 800 Washington Street, Box 245, Boston, MA 02111 USA; 2 Novogen, Sydney, Australia

**Keywords:** Chemo-resistant, Phenoxodiol, Cantrixil, Ovarian cancer, Flavonoids, Super-benzopyran

## Abstract

**Purpose:**

Recurrent, chemo-resistant ovarian cancer is thought to be due to a subgroup of slow-growing, drug-resistant cancer cells with stem-like properties and a high capacity for tumour repair. Cantrixil targets this sub-population of cells and is being developed as an intraperitoneal therapy to be used as first-line therapy in combination with carboplatin for epithelial ovarian cancer. The studies presented here justify further development.

**Methods:**

A GLP dog CV study using a 4 × 4 Latin Square Crossover study was conducted using telemetric ECG recordings from dogs post IP administration to assess for cardiac abnormalities. Mutagenic potential was assessed using the bacterial reverse mutation assay. Clastogenicity was assessed by determining micronuclei formation in the bone marrow of SPF Arc(S) Swiss mice dosed at clinical concentrations. TRX-E-002-1 toxicology was evaluated in GLP-compliant MTD and 28-day repeat-dose studies in rats and dogs.

**Results:**

In vitro TRX-E-002-1 has potent cytotoxic activity against human cancer cells including CD44+/MyD88+ ovarian cancer stem cells. TRX-E-002-1 increased phosphorylated c-Jun levels in these cancer cells resulting in caspase-mediated apoptosis. In vivo, Cantrixil was active in a model of disseminated ovarian cancer as a monotherapy and in combination with Cisplatin. Cantrixil was active as maintenance therapy in a model of drug-resistant, recurrent ovarian cancer and in an orthotopic model of pancreatic cancer.

**Conclusions:**

In animals, this clinical formulation and route of administration of Cantrixil demonstrated acceptable activity, safety pharmacology, genotoxicity and toxicology profile and constituted a successful Investigational New Drug application to the US Food and Drug Administration.

## Introduction

With an age-standardised incidence of 9.4 women developing epithelial ovarian cancer (EOC) per 100,000 in the USA equating to 22,500 women developing the disease annually, approximately 14,200 of those women will die each year [1–3]. These statistics show that EOC accounts for the greatest number of deaths from all reported gynaecologic malignancies [4–7]. Early detection of the disease is critical for improved survival; however, due to non-specific symptomology and the lack of an effective screening marker with high sensitivity and specificity, many patients present at later stages of the disease (International Federation of Gynaecology and Obstetrics (FIGO) stage III) where the 10-year survival rate is poor (10–20%) [8]. Advances in therapeutic regimens employing traditional cytotoxic chemotherapy that utilise intraperitoneal delivery and dose-dense administration are improving response rates, as are targeted agents like bevacizumab, but these treatments fail to improve overall survival [9]. Moreover, patients often develop resistance to chemotherapy thought to be due to the presence of residual cancer stem cells in tumour niches, hence the urgent need to identify novel therapeutics that target these slow-growing, highly drug-resistant stem-like cancer cells.

**Fig. 1:**
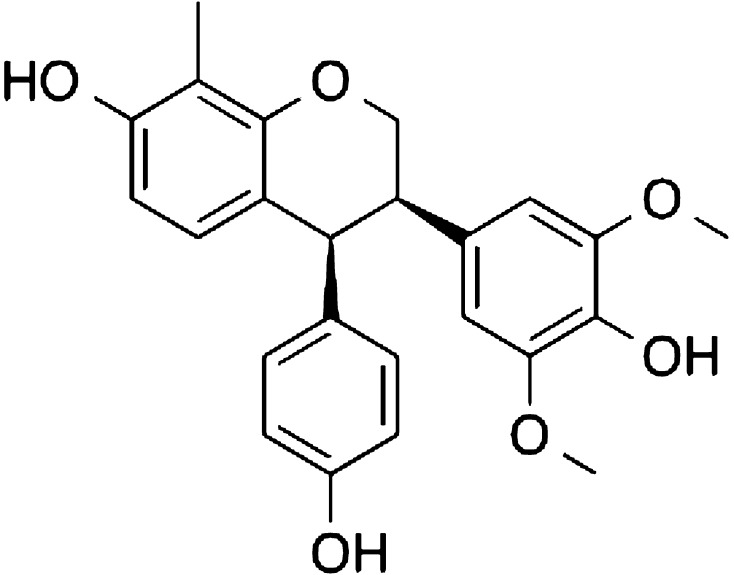
Chemical structure of TRX-E-002-1 (Cantrixil)

Our lead drug candidate, TRX-E-002-1 (Fig. 1), is the active enantiomer of a super-benzopyran molecule that has been identified as having potent pan anti-cancer activity against a broad range of cancer phenotypes. It was selected particularly for its potent cytotoxicity against the two main sub-populations of ovarian cancer: chemo-resistant CD44+/MyD88+ ovarian cancer stem cell (OCSC) clones as well as in chemo-sensitive CD44−/MyD88− ovarian cancer cell (OCC) lines and potent activity in in vivo model of disseminated ovarian cancer.

## Genesis of the super-benzopyran drug class

Plant-derived phytochemicals include several broad classes of compounds including thiols (such as the isothiocyanates), terpenes (such as carotenoids and non-carotenoids) and phenols (including flavonoids and non-flavonoids). Epidemiological studies have shown that those countries with populations that consume diets high in phytochemicals typically have a lower incidence of cancer in the general population. Screening of phytochemicals against malignant cells have demonstrated that this family of molecules have broad pleiotropic anti-cancer effects that potentiate their ability at inhibiting cell proliferation, angiogenesis and, inducing mitotic arrest and apoptosis [10]. The anti-cancer properties of dietary phytochemicals may also be attributable to their epigenetic properties, including changes in DNA methylation patterns, histone modifications and miRNA expression levels [11]. Furthermore, phytochemicals have been shown to modulate signalling pathways (i.e. Hedgehog, Wnt/β-catenin and Notch) in cancer stem cells which display enhanced DNA damage repair mechanisms, amplified anti-apoptotic activity, enhanced xenobiotic efflux and skewed production of certain pre-inflammatory cytokines [12].

Phytochemicals such as the flavonoid genistein are polyphenolic compounds found in plants characterised by a simple benzopyran structure. They are known to possess anti-tumour properties by inducing mitotic arrest and apoptosis. In addition, genistein can inhibit Notch signalling and mammosphere formation of breast cancer cells [13].

**Fig. 2:**
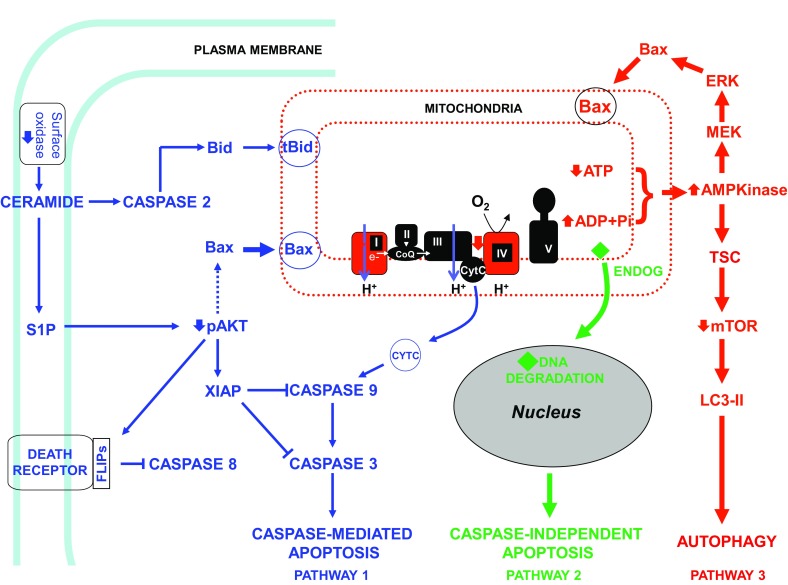
Mechanisms of cell death induced by benzopyran compounds by minor chemical modifications of the benzopyran scaffold. Minor modifications of the benzopyran scaffold alter the mechanisms of cell death. Phenoxodiol induces classic caspase-mediated apoptosis caused by inhibition of a surface NADH oxidase and disruption of the sphingomyelin cycle resulting in caspase-mediated apoptosis (pathway 1); ME-128, NV-143 and ME-344 (previously NV-128) uncouple mitochondrial oxidative phosphorylation resulting in autophagy mediated by increased AMP kinase due to changes in the ATP/ADP ratio and mTOR disruption and cell death induced by endonuclease G translocation to the nucleus that proceeds in the presence of a pan-caspase inhibitor (pathway 2 and 3); triphendiol (NV-196) induces both caspase-mediated and caspase-independent cell death (pathways 1 and 2). Mitochondrial respiratory chain complexes (I, II, III, and IV), along with complex V (ATP synthase), function together in mitochondrial oxidative phosphorylation and are shown in the inset

Medicinal chemists exploited the pan anti-cancer activity of the benzopyran pharmacophore found in genistein to develop the first anti-cancer drug candidate, phenoxodiol (idrinoxol) [14]. While demonstrating anti-cancer activity in animals in vivo as a monotherapy, the primary effect of phenoxodiol was its ability to enhance the cytotoxic effects of paclitaxel and cisplatin in taxane- and platinum-sensitive and -resistant cancer cells. Phenoxodiol was granted Investigational New Drug (IND) status by the US Food and Drug Administration (FDA) in February 2001 as an intravenous formulation and in June 2003 as an oral formulation. The intravenous formulation of phenoxodiol underwent three Phase I trials in multiple cancer types in Australia and in the USA [15]. Further developments in medicinal chemistry achieved progressively greater structural complexity of the simple benzopyran drug technology platform advancing from phenoxodiol, to triphendiol to NV-128 and then to ME-344, resulting in progressive log-fold increases in anti-cancer activity. Along with the increased potency came different mechanisms of cell death, with phenoxodiol causing caspase-dependent apoptosis via XIAP uncoupling, triphendiol causing both caspase-dependent and -independent apoptosis, and NV-128, NV-143 and ME-344 killing cancer cells by the uncoupling of mitochondrial oxidative phosphorylation resulting in catastrophic autophagy and caspase-independent apoptosis (see Fig. 2) [14, 16–18].

ME-143 (previously NV-143) was granted IND status for an intravenous formulation in August 2011 and has recently completed a Phase I trial [19]. An intravenous formulation of ME-344 was granted IND status in May 2012. ME-344 has recently completed a Phase I first-in-man trial [20] and is about to commence an investigator-sponsored study of ME-344 in combination with the VEGF inhibitor bevacizumab (Avastin^®^) in HER2-negative breast cancer in 2016 (MEI Pharma website).

Using Novogen’s proprietary Versatile Approach to Library-based Iterative Design (VAL-ID), we have created a library of benzopyrans of even greater complexity through the addition of a range of chemical moieties that were previously technically impossible (termed super-benzopyrans, SBPs). This strategy is based around the design, synthesis and evaluation of targeted small-molecule libraries and has proven to be a rapid and robust method of identifying lead compounds. Several discrete sub-families of these SBPs have been identified on the basis of chemical composition, with up to thousands of potential analogues possible per patent sub-family. Without an initial target or docking studies, the discovery program has followed a ligand-based design strategy. This approach has allowed the generation of detailed structure activity relations (SAR’s) of the various SBP libraries. This allowed the definition of key structural requirements for enhanced activity, metabolic stability and pharmaceutical properties. We have created first- and second-generation drug candidates that exhibit significant activity against several types of cancer, including ovarian, both in vitro and in vivo. The latest drug discovery program has yielded a number of lead candidate compounds. The first of these compounds, TRX-E-002-1, is the subject of this review.

## TRX-E-002-1

### Pharmacology

The chemical name of TRX-E-002-1 is (+) cis-4-(para-hydroxyphenyl)-7,4′-dihydroxy-3′,5′-dimethoxy-8-methylisoflavan (Fig. 1). The chemical formula is C_24_H_24_O_6_, and its molecular weight is 408.2. Synthesis of the active pharmaceutical ingredient yields a racemic mixture of two enantiomers, the active enantiomer is termed TRX-E-002-1 and the inactive enantiomer is termed TRX-E-002-2. The final step in the manufacture of TRX-E-002 is a catalytic heterogenous hydrogenation. This reaction is a stereospecific reaction that generates only the *cis*-oid stereoisomers around the core pyran ring. The two *cis*-oid stereoisomers are resolved into the two enantiomers via chiral chromatographic techniques. TRX-E-002-1 is the biologically active enantiomer and active ingredient of the drug product Cantrixil which is formulated in sulfobutylether β-cyclodextrin (Dexolve™).

Efficacy of TRX-E-002-1 has been demonstrated against a range of human cancer cell lines in vitro. TRX-E-002-1 showed broad cytotoxic activity against ovarian, prostate and lung cancer cells, with IC_50_ values ≤0.1 μM. Activity in pancreatic and colorectal cancer cells and glioblastoma cells was more variable (Table 1).

**Table 1:** Cytotoxic activity of TRX-E-002-1 in human cancer cell lines

Cancer type	Cell line	IC_50_ (μM)^a^
Ovarian	SK-OV-3	0.028 ± 0.003
	SK-OV-3	0.109 ± 0.026
	JAM	0.065 ± 0.002
	OVCAR-3	0.023 ± 0.006
Prostate	DU145	0.041 ± 0.014
	PC3	0.096 ± 0.077
	C4-2B	0.014 ± 0.009
Lung	A549	0.058
Pancreatic	Panc-1	0.467 ± 0.378
	ASPC1	0.227 ± 0.055
	MiaPaCa2	3.72 ± 3.65^b^
Colorectal	HT-29	1.765 ± 1.385
	LOVO	0.084
	LOVO	0.045 ± 0.005
Glioblastoma	A172	0.051 ± 0.002
	U87MG	0.205
	U87MG	0.1 ± 0.07

^a^Concentration for 50% inhibition of cell growth (IC_50_) after incubation with TRX-E-002-1 for 72 h. Results are mean values ± standard error of mean (SEM) from duplicate experiments for, or from duplicate wells on triplicate plates (*n* = 6)

^b^Duplicate experiments gave discrepant results, with respective IC_50_ values of 0.067 and 7.37 μM

Tumour recurrence post-chemotherapy is caused by the regrowth of the surviving innately drug-resistant cancer stem cells that remain in tumour niches during and post-chemotherapy. The expansion of this chemo-resistant cancer cell population, coupled with upregulation of pro-survival pathways due to drug pressure, is thought to be responsible for the development of chemo-resistant recurrent disease [21]. Further, the inherent heterogeneity of ovarian tumours consists of epithelial cells with varying potential for stemness, and a population of cancer cells with varying mesenchymal status and invasiveness potential [22]. Therefore, to improve survival, a practical approach is the use of novel therapies that can induce cell death in these various subtypes of ovarian cancer cells. TRX-E-002-1 was assessed against ovarian cancer stem cell line OCSC2, characterised by their expression of stem cell markers including CD44 and MyoD [22]. A major characteristic of these cells is that they are drug resistant, able to form self-renewing spheroids in non-adherent culture, and recreate disseminated drug-resistant ovarian tumours that have a high degree of heterogeneity similar to the clinical profile observed in patients when inoculated into the peritoneal cavity of an immune-compromised mouse [22–24]. TRX-E-002-1 is able to induce cell death in chemo-resistant CD44+/MyD88+ OCSC clones (Table 1) and chemo-sensitive CD44−/MyD88− ovarian cancer cell lines when grown separately or in co-cultures which mimic tumour heterogeneity. We also demonstrated that TRX-E-002-1 enhanced the effectiveness of cisplatin in co-culture studies and promoted the degradation of tumour spheroids.

In vivo, TRX-E-002-1, formulated in 20% SBECD, was active in a disseminated ovarian cancer model and a recurrent ovarian cancer model in which tumour recurrence is investigated following initial treatment of the OCSC2-inoculated mice with paclitaxel. In both models, TRX-E-002-1 inhibited tumour growth following once daily intraperitoneal administration.

In the disseminated ovarian cancer model, once daily intraperitoneal administration of 100 mg/kg racemic TRX-E-002 or the active enantiomer TRX-E-002-1 in 20% SBECD for 13–14 days significantly inhibited tumour growth and reduced excised tumour weight at termination by 50–72%. In a dose-ranging study, the most effective dose regimen was 100 mg/kg compared with either a lower daily dose (50 mg/kg/day) or a higher dose administered intermittently (150 mg/kg 3 times weekly). All three regimens were well tolerated.

In the recurrent ovarian cancer model, once daily intraperitoneal administration of 100 mg/kg TRX-E-002-1 in 20% SBECD for 4 weeks inhibited tumour growth and reduced terminal tumour burden by 77%. Additionally, TRX-E-002-1 was more effective than paclitaxel administered 12 mg/kg twice weekly.

The combination of TRX-E-002-1 and cisplatin was investigated in the murine model of disseminated ovarian cancer. Tumour growth during the 16-day treatment period was significantly inhibited by TRX-E-002-1 (100 mg/kg once daily), cisplatin (5 mg/kg once weekly) or TRX-E-002-1 in combination with cisplatin. During the 5-week post-treatment period, tumour growth was decreased in both monotherapy groups, and to a significantly greater extent in the combination treatment group. By decreasing tumour growth kinetics in recurrent disease in this model, TRX-E-002-1 treatment resulted in lower tumour burden. Given that residual disease has been shown to directly correlate with survival and is a prognostic factor in ovarian cancer, TRX-E-002-1 has the potential to increase survival by reducing the rate of recurrent disease [25].

Together, these data demonstrate that TRX-E-002-1 is active against ovarian cancer stem cells and tumour spheres. It has the potential to prevent the formation of niche cancer stem cells within tumours that survive chemotherapy thereby preventing tumour recurrence. Cantrixil may enhance the effectiveness of standard-of-care cytotoxic therapeutics like cisplatin thereby enhancing overall survival benefits [25].

**Fig. 3:**
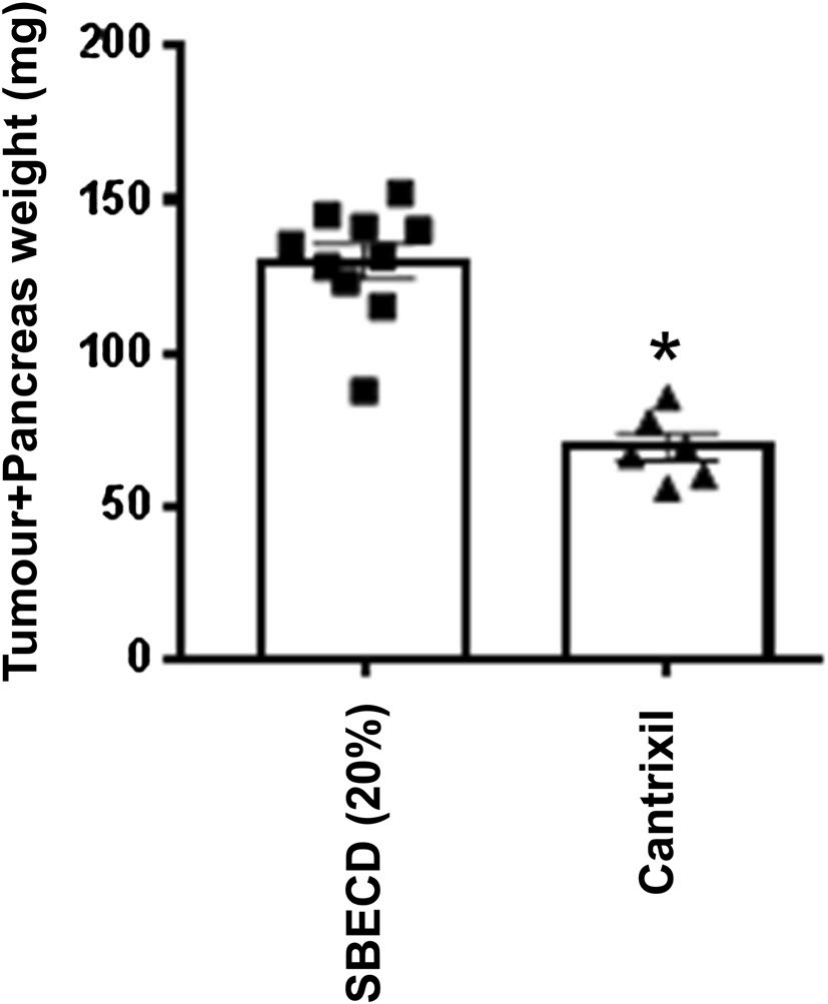
Efficacy of TRX-E-002-1 in an orthotopic model of pancreatic cancer. Female NOD-SCID mice were inoculated directly into the pancreas with PANC-1 human pancreatic epithelioid adenocarcinoma cells transfected with luciferase gene. Tumours were allowed to establish prior to treatment initiation. Animals were dosed with TRX-E-002-1 100 mg/kg i.p. for 18 consecutive days over which time tumour growth was monitored using bioluminescent imaging. Following euthanasia at the predetermined end-point, tumours were excised and weighed. Final tumour (plus pancreas) weights excised at the terminal time point. *p < 0.001

In a mouse model of pancreatic cancer (human Panc-1 pancreatic tumour cells implanted orthotopically into female NOD-SCID mice), once daily intraperitoneal administration of TRX-E-002-1 100 mg/kg in 20% SBECD significantly reduced terminal pancreatic tumour burden (Fig. 3). These data provide in vivo evidence that TRX-E-002-1 may have clinical application against other abdominal tumours.

The role of caspases and intracellular signalling pathways in the anticancer activity of TRX-E-002-1 was investigated in OCSC2 ovarian cancer stem cells. The activity of caspases 3/7 and 9 was markedly increased by treatment with TRX-E-002-1. Activation of caspase 3/7 by TRX-E-002-1 was also demonstrated in cell lines representative of colon, lung and brain cancer, indicating that TRX-E-002-1 is able to induce cell death across a range of cancer types (data not shown). Western blots showed decreased levels of inactive caspase 2 at both concentrations of TRX-E-002-1, implying proteolytic conversion of this precursor to active caspase 2. TRX-E-002-1 also modulated phosphorylation of intracellular signalling molecules, with decreased levels of pERK (phosphorylated extracellular signal-regulated kinase) and increased levels of phosphorylated c-Jun (p–c-Jun). Activated p–c-Jun levels increased progressively over the 24-h incubation period in response to both concentrations of TRX-E-002-1 tested. The results indicate that TRX-E-002-1-induced death of OCSC2 cells is associated with the phosphorylation/activation of c-Jun and down-regulation of pERK. Increased p–c-Jun and decreased pERK levels were observed following incubation of OCSC2 cells after only 2-h’ exposure, indicating these changes are one of the earliest effects of TRX-E-002-1 treatment.

Further evidence for the role of c-Jun phosphorylation in the anti-tumour efficacy of TRX-E-002-1 comes from an experiment in OCSC2 cells incubated with the selective inhibitor of c-Jun N-terminal kinase, SP600125 [25]. Western blotting confirmed that 10 μM SP600125 inhibited TRX-E-002-1-induced phosphorylation of c-Jun without affecting phosphorylation of ERK. SP600125 blocked the anti-tumour activity of 0.245 μM TRX-E-002-, indicating that the cytotoxic activity in OCSC2 cells is dependent on phosphorylation of c-Jun. Similar results were observed using SiRNA to knock down c-Jun [25].

**Fig. 4:**
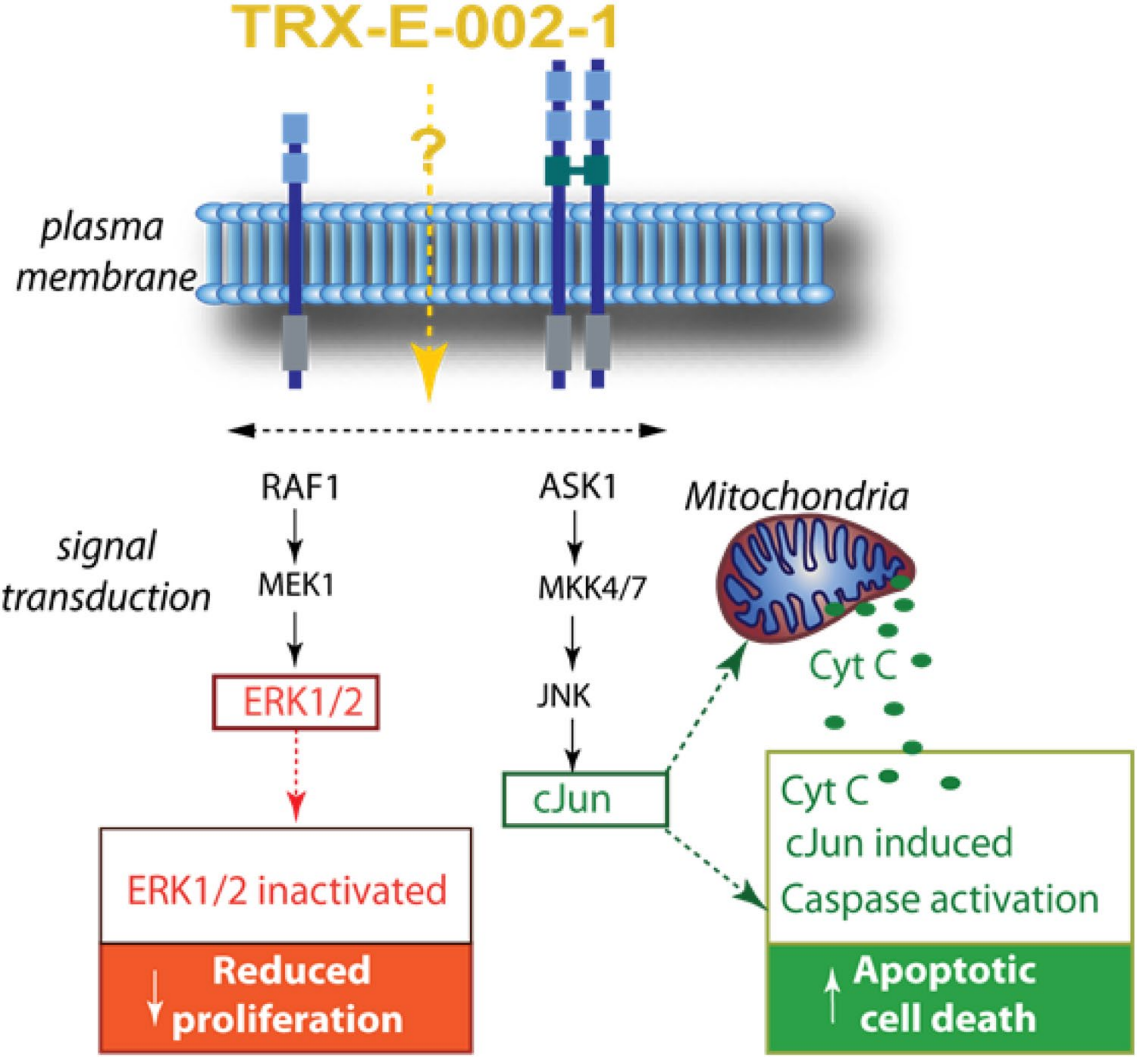
Simple schematic overview of TRX-E-002-1-induced changes in signalling pathways known to influence cell proliferation and death

Further studies are required to determine the initial trigger of TRX-E-002-1 induced apoptosis. Our preliminary mechanism of action studies demonstrate that TRX-E-002-1 is modulating both pro-survival and pro-death pathways resulting in caspase-mediated apoptosis (Fig. 4).

### Safety pharmacology

Safety pharmacology studies have shown that TRX-E-002-1 does not affect surface expression of wild-type human ether-à-go-go-related gene (hERG), indicating that it does not affect hERG trafficking. However, at concentrations of 0.1-10 μM, the drug increased surface expression of the hERG single mutant (hERG-SM), identifying it as a potential blocker of hERG channel activity. A patch-clamp electrophysiological assay in hERG-transfected mammalian cells showed that TRX-E-002-1 concentration-dependently inhibited hERG current with IC_50_ of 17.0 μM. Potential cardiovascular responses to intraperitoneal injection of TRX-E-002-1 were investigated in a GLP-compliant telemetry study in beagle dogs. Four male dogs received vehicle (20% SBECD) or TRX-E-002-1 (1, 10 or 30 mg/kg) on four separate occasions in a Latin Square design. Blood pressure, heart rate, ECG parameters and body temperatures were continuously recorded from 30 min before dosing until at least 24 h after each dose. Electrocardiographic (ECG) readings in dogs showed no effect of TRX-E-002-1 on the QTc interval following intraperitoneal administration at 1, 10 or 30 mg/kg. The dog study showed slight, transient increases in blood pressure and heart rate at 10 and/or 30 mg/kg, but these effects were not considered adverse [26].

### Pharmacokinetic drug–drug interactions

The potential for pharmacokinetic drug interactions resulting from inhibition of drug-metabolizing cytochrome P450 enzymes was investigated in human hepatic microsomes in vitro. TRX-E-002-1 inhibited multiple cytochrome P450 drug-metabolizing enzymes, including CYP2C9, CYP2C8, CYP2C19, CYP2B6, CYP3A4, CYP2D6, CYP2A6 and CYP1A2. IC_50_ values ranged from 1.5 to 75 μM (612–30,600 ng/mL). Metabolism-dependent inhibition was observed for CYP2C8, CYP3A4 and CYP1A2. These results suggest that caution is warranted when administering Cantrixil in combination with drugs metabolised by cytochrome P450 enzymes until further information is available. In particular, the potential interaction between Cantrixil and paclitaxel, a CYP2C9 substrate frequently used in the treatment of ovarian cancer, needs to be considered.

Potential induction of human drug-metabolizing cytochrome P450 enzymes by TRX-E-002-1 was also investigated in human hepatocytes in vitro (VPT3079/2015). No evidence of CYP1A2 induction was observed. Equivocal evidence for induction of CYP2B6 and CYP3A4 was seen in microsomes from one of three donors. Based on these results, TRX-E-002-1-mediated activation of pregnane X receptor (PXR, responsible for CYP3A4 induction) and/or constitutive androstane receptor (CAR, responsible for CYP2B6 induction) cannot be excluded [26].

### TRX-E-002-1 pharmacokinetics

#### Absorption, distribution metabolism and excretion

The pharmacokinetics of TRX-E-002-1 was evaluated in male female Sprague–Dawley rats following a single intraperitoneal injection of 100 mg/kg TRX-E-002-1 in 20% SBECD vehicle. Absorption was rapid, with *C*_max_ mean plasma concentrations reached 0.25/1 h after dosing in female/male rats. Higher *C*_max_ levels were observed in males. Mean plasma levels decreased to below the limit of quantification by 24 h (not shown). The AUC_0−∞_ value was 29,537 and 44,702 ng h/mL in female and male rats, respectively (Table 2). High tissue levels were observed 0.5–6 h after dosing, with levels higher than the plasma concentration in pancreas, ovaries, large intestine, skin, kidneys, liver, stomach and adrenal glands (not shown). Treatment of plasma and tissue samples with glucuronidase indicated the presence of low levels of a glucuronide conjugate of TRX-E-002-1 (not shown). Other metabolic pathways have not yet been investigated. Plasma elimination half-life was approximately 1.3 and 1.9 h. in female and male rats, respectively. Parent drug was excreted in the urine and faeces, and high levels of the glucuronide metabolite were found in the faeces.

**Table 2:** Pharmacokinetics of TRX-E-002 in rat and dog plasma after IP injection

Parameter	Rat (*F*)	Dog (*M* + *F*)			Dog (*M* + *F*)
Dose (mg/kg)	100	10	30	100	10
*T*_max_ (h)	0.5	2.0	2.0	1.25	2
*C*_max_ (ng/mL)	8355	626	1925	6795	425
*T*_½_ (h)	~2.5	2.6	2.5	2.2	2.6
AUC_last_ (ng h/mL)	29,396	4390	8770	58,500	1630
AUC_0−∞_ (ng h/mL)	40,600	4395	11,300	58,550	1633

Absorption of TRX-E-002-1 in beagle dogs was investigated following dosing on Day 1 of a 14-day pilot toxicity study. Plasma samples were collected and analysed following intraperitoneal administration of TRX-E-002-1 at 10, 30 or 100 mg/kg. Absorption was rapid, with peak plasma concentration observed between 0.5 and 2 h. Mean *C*_max_ values were roughly proportional to dose (626, 1935, 6795 ng/mL at 10, 30 and 100 mg/kg, respectively). TRX-E-002-1 was still detectable in most dogs 24 h after dosing, and mean AUC_0−last_ values were 4390, 8770 and 58,500 ng h/mL, respectively. Plasma elimination half-life in individual animals ranged from 2.0 to 2.7 h, independently of dose. Mean AUC_0−∞_ values were 4395, 11,300 and 58,550 ng h/mL, respectively. Similar plasma pharmacokinetic values were observed in a second study in which four dogs received a 10-mg/kg intraperitoneal dose of TRX-E-002-1. In the latter study, TRX-E-002-1 concentrations in gastrointestinal tissues 24 h after dosing were at least 30 times higher than those in plasma, with the highest concentrations being observed in the colon. However, by 72 h, TRX-E-002-1 levels were close to or below the limit of quantification (Table 2).

#### TRX-E-002 ADME interspecies comparison

In female rats, plasma concentrations of unconjugated TRX-E-002-1 were approximately 90% of the total levels (parent + glucuronide) up to 6 h after dosing, indicating that glucuronidation is a minor metabolic pathway in female rats. Tissue concentrations of total drug (parent + glucuronide) were also similar to those in plasma except in liver, where they were 2–3 times higher.

In beagle dogs, plasma concentrations of unconjugated TRX-E-002-1 at early time points (1–4 h’ post-dose) were 76–93% of the total levels (parent + glucuronide). At 8 h, the plasma concentrations of the glucuronide metabolites were similar to those of parent drug. By 24 h’ post-dose, the concentrations of TRX-E-002-1-glucuronide in plasma and gastrointestinal tissues (except colon) were 2–12 times higher than those of the parent drug. The increased levels of TRX-E-0021-glucuronide relative to those of parent drug at 24 h post-dose may reflect slower elimination of the glucuronide metabolites.

Dose proportionality has not been investigated in rats, but linear pharmacokinetics was observed in dogs. Elimination half-life appeared to be about 2.5 h in rats, and 2.2–2.6 h in dogs.

#### Excretion

Excretion of TRX-E-002-1 was investigated in female Sprague–Dawley rats that received a single intraperitoneal injection of TRX-E-002-1 100 mg/kg in 20% SBECD. High levels of parent drug were measured in urine up to 24 h after dosing and in faeces up to 48 h after dosing. The glucuronide conjugate, measured as the increase in TRX-E-002-1 following treatment of the samples with beta-glucuronidase, was found at levels similar to those of parent drug in faeces (rectal contents) up to 2 h after dosing, but was barely detectable in urine. Total amounts of parent drug and glucuronide excreted in faeces and urine were not calculated, so that the relative contributions of renal and biliary excretion pathways cannot be evaluated.

### TRX-E-002-1 toxicology

Cantrixil [TRX-E-002-1 formulated in sulfobutylether-beta-cyclodextrin (SBECD)] is being developed as an intraperitoneal therapeutic for abdominal cancers. All animal toxicity studies have therefore been completed using intraperitoneal administration. The pivotal repeat-dose studies in rats and dogs were 28 days in duration; both were compliant with Good Laboratory Practice (GLP) regulations. Dose selection in the pivotal studies was supported by non-GLP single-dose and 14-day repeat-dose studies in both species. Non-GLP single-dose intraperitoneal toxicity studies were conducted in rats and dogs as dose-ranging studies to determine the maximum tolerated dose. Toxicokinetic assessments were included in all four repeat-dose toxicity studies. In the two dose-ranging studies, plasma concentrations of TRX-E-002-1 on Day 14 were determined. In Sprague–Dawley rats, Cantrixil was well tolerated following single intraperitoneal doses of 50 and 100 mg/kg, but deaths were observed at 150 mg/kg (females) and 200 mg/kg (both sexes). Test article-related clinical signs consisted of rough fur, stained fur, hunched posture, lethargy, swelling of the abdomen, discoloured faeces, liquid faeces, soft faeces, reduced faeces and anogenital staining. Many of these signs were also observed at 100 mg/kg, and faecal reduction was observed at all doses (≥12.5 mg/kg). Body weight gain was reduced at 150 and 200 mg/kg. As a single administration, the maximum tolerated dose was considered to be 50 mg/kg for both sexes. Based on these observations, 50 mg/kg/day was selected as the high dose for the 14-day repeat-dose intraperitoneal toxicity studies in rats.

In the rat 14-day pilot study, male and female SD rats were dosed once daily with intraperitoneal injections of vehicle (1:4 dilution of 20% SBECD in 0.9% saline) or TRX-E-002-1 (5, 15 or 50 mg/kg) for 14 days. Deaths were observed at 15 mg/kg/day (one female) and 50 mg/kg/day (9/11 males and 8/11 females), and dosing of the remaining high-dose animals was terminated on Day 6. Clinical signs at all dose levels were reduced and/or liquid faeces, and staining of the fur of the forelimbs or around the eyes, nose and snout. Anogenital staining, rough fur, hunched posture, reduced activity and soft faeces were observed at 15 and/or 50 mg/kg/day. Body weight gain was dose-dependently reduced at doses ≥5 mg/kg/day in males and ≥15 mg/kg/day in females. Clinical laboratory assessments at 5 and/or 15 mg/kg/day showed decreased erythrocytic parameters, increased urea, and decreased albumin and total protein. Macroscopic pathology findings were limited to the 50 mg/kg/day group and included small testes, small thymus and small spleen. Organ weight analysis showed reduced weights of thymus, testes and ovaries at 5 and/or 15 mg/kg/day. Treatment-related histopathological changes were observed in testes (degeneration of spermatogenic elements lining the seminiferous tubules at all dose levels), adrenal glands (vacuolation of the zona fasciculata at 15 and 50 mg/kg/day) and spleen (depletion of lymphocytes). Toxicokinetic results on Day 14 at the no-observed-adverse-effect level (NOAEL) of 5 mg/kg/day were plasma *C*_max_ was 909 and 257 ng/mL in males and females, respectively, and corresponding AUC_last_ values of 1870 and 665 ng h/mL.

In the rat GLP 28-day repeat-dose study, male and female SD rats were dosed once daily with intraperitoneal injections of vehicle (1:20 dilution of 20% SBECD in 0.9% saline) or TRX-E-002-1 (0.5, 3 or 10 mg/kg) for up to 28 days. TRX-E-002-1 peak plasma concentrations were observed 0.25 – 0.5 h after dosing, except for mid-dose females on Day 1 (*T*_max_ = 1 h). Estimated plasma elimination half-life was similar across sexes and doses ranging from 0.7 to 1.4 h on Day 1 and tended to be slightly prolonged at the mid- and high-doses on Day 28 (range = 0.9–2.3 h). Mean plasma TRX-E-002-1 concentrations were roughly proportional to dose and were generally higher in males than in females. *C*_max_ values for the 0.5, 3 and 10 mg/kg doses on Day 1 were 35.4, 181 and 780 ng/mL, respectively, in males, and 33.4, 150 and 535 ng/mL, respectively, in females. AUC_last_ values on Day 28 tended to be higher than those on Day 1, except in low-dose males and high-dose females (Table 2), indicating slight accumulation of TRX-E-002-1 with daily dosing.

No deaths were observed during the study. No clinical signs were observed at the low dose of 0.5 mg/kg/day. At 3 mg/kg, reduced quantity of faeces was observed in both sexes during the first week of treatment. At 10 mg/kg, reduced quantity of faeces was observed during the first 1 or 2 weeks in all animals. During the recovery period, faeces were normal, although anogenital staining persisted up to 4 days after cessation of dosing in one high-dose male.

Body weight gain was reduced over Days 1–28 in high-dose males, and during the first treatment week in mid-dose males and high-dose females. Mean body weights at the end of the dosing period were 99.5, 95.7 and 89.2% of control values in males at 0.5, 3 and 10 mg/kg/day, respectively; corresponding values in females were 99.8, 97.8 and 97.8%, respectively. Mean body weight of the high-dose male group recovered to 93.6% of control value after the 14-day recovery period. Food consumption was significantly reduced throughout the dosing period in high-dose male and females, but was similar to control values during the recovery period. Ophthalmoscopy examination towards the end of the treatment period showed no treatment-related abnormalities.

Treatment-related macroscopic abnormalities at the terminal necropsy were limited to distention of the caecum and/or colon in both sexes at 10 mg/kg/day, and obstruction of the colon in 3/10 males at this dose level. No macroscopic findings were present after the recovery period. Organ weight analysis showed decreased absolute and relative testicular weights at 3 and 10 mg/kg/day (associated with decreased absolute epididymal weight at 10 mg/kg/day), and decreased weights of salivary glands (absolute and relative to brain weight) in both sexes at 3 and 10 mg/kg/day. Thymic weight was reduced in males at 3 and 10 mg/kg/day and was increased in females at 10 mg/kg/day. Testicular and epididymal weights were still reduced in the 10 mg/kg/day group after the recovery period.

Serum biochemical assessments at the end of the 28-day treatment period showed slight (≤8%) decreases in total protein, albumin and globulin in males and females at 10 mg/kg/day; these changes were statistically significant only for total protein and albumin in males. Similar changes were also seen after the 14-day recovery period (significant in males only). Haematological, coagulation and urinalysis assessments showed no treatment-related changes.

Histopathological examination showed treatment-related lesions in testes, epididymides and salivary glands. In the testes, degeneration of spermatogenic elements in the seminiferous tubules was observed with dose-dependent severity at 3 and 10 mg/kg/day. At 10 mg/kg/day, multifocal degeneration was mild, and marked diffuse degeneration was observed in two cases. Sperm degeneration was observed in the epididymides of all males in the same two dose groups, and was minimal to mild at 3 mg/kg/day, and moderate to marked at 10 mg/kg/day.

In summary, the major toxicity finding in rats dosed once daily for 28 days with TRX-E-002-1 was degeneration of seminiferous tubules in the testes and of sperm in the epididymides at the high dose of 10 mg/kg/day. Other findings including transient faecal changes, reductions in weight gain and food consumption, serum biochemical changes, macroscopic lesions in the large intestine and atrophy of the salivary glands were considered non-adverse. The NOAEL was considered to be 3 mg/kg/day in male rats and 10 mg/kg/day in females. The greater sensitivity of male rats compared with females would appear to be associated with higher cumulative systemic exposure over the 28-day repeat-dose period.

For the acute dog MTD study, male and female beagle dogs were dosed with ascending doses of Cantrixil (10, 20, 40, 80 and 200 mg/kg) as a single intraperitoneal injection. While no deaths were observed, treatment-related clinical signs at the highest dose were faecal changes (soft, liquid, mucoid, yellow discoloured, decreased), yellow emesis, thin appearance, lethargy and reduced activity. Body weight was not affected. Based on these observations 100 mg/kg/day was selected as the high dose for the 14-day repeat-dose intraperitoneal toxicity studies in dogs.

For the dog 14-day pilot study, male and female beagle dogs were dosed once daily with intraperitoneal injections of vehicle (1:2 dilutions of 20% SBECD in 0.9% saline) or Cantrixil (10, 30 or 100 mg/kg) for 14 days. The doses were administered via an implanted catheter. Treatment-related deaths were observed at 30 mg/kg/day (female on Day 11) and 100 mg/kg/day (both sexes, Day 5). Treatment-related clinical signs at all dose levels included soft and liquid faeces, emesis and reduced activity. Body weights were decreased at 10 and 30 mg/kg/day. Necropsy findings in the animals that died included dark red discoloration in the gastrointestinal tract associated with microscopic finding of diffuse congestion of the submucosa. Similar findings were observed in the TRX-E-002-1-treated animals at the terminal sacrifice. Toxicokinetic measurements on Day 1 showed peak plasma concentrations at 30–120 min after dosing. No gender differences were observed, and mean *C*_max_ values for combined sexes were proportional to dose: 626, 1935 and 6795 ng/mL at 10, 30 and 100 mg/kg/day, respectively. Mean AUC_last_ values were also roughly proportional to dose: 4390, 8770 and 58,500 ng h/mL, respectively. Plasma concentrations in surviving dogs on Day 14 were lower than those on Day 1, with AUC_last_ values of 1470 and 6270 ng h/mL at 10 and 30 mg/kg/day, respectively. The NOAEL in this study was 10 mg/kg/day.

For the dog GLP 28-day repeat-dose study, beagle dogs received intraperitoneal injections of vehicle (3:20 dilution of 20% SBECD in 0.9% saline) or Cantrixil (1, 10 or 30 mg/kg) three times per week for 4 weeks via an implanted catheter. Blood samples were collected from all dogs at 0.25, 0.5, 1, 2, 4 and 24 h after dosing on Days 1 and 26. Peak plasma concentrations were observed 1–2 h after dosing, and group mean *T*_max_ values ranged from 1.0 to 1.8 h on Day 1, and from 1.2 to 1.5 h on Day 26. Estimated plasma elimination half-life was similar across sexes, doses and sampling days and ranged from 1.2 to 2.6 h. Mean *C*_max_ and AUC_last_ values were similar in males and females and were greater than proportional to dose. Mean *C*_max_ values in males/females on Day 1 were 28.1/30.4, 497/453 and 1360/2060 ng/mL for the 1, 10 and 30 mg/kg doses, respectively. Corresponding mean AUC_last_ values on Day 1 were 71.4/80.5, 2810/2260 and 10,500/11,000 ng h/mL, respectively, for males/females. Mean *C*_max_ and AUC_last_ values on Day 26 were similar to that on Day 1, indicating no accumulation of TRX-E-002-1 with three times weekly dosing.

Treatment-related clinical signs observed in males and females at 10 or 30 mg/kg included emesis, and soft or liquid faeces. Mean body weight gain and food consumption were slightly reduced in male dogs at 30 mg/kg. Food consumption was affected to a lesser extent in females at 30 mg/kg and was not associated with discernible effect on body weight gain. Ophthalmological examination and electrocardiography and in Week 4 did not show treatment-related abnormalities.

Haematological assessment showed slight but significant increases in total leucocyte in males and females at 10 and 30 mg/kg, with a similar trend in males at 1 mg/kg. The differential leucocyte count showed increased absolute numbers of neutrophils and monocytes. Similar changes were apparent but less severe in high-dose males and females after the 2-week recovery period. Coagulation parameters, serum chemistry, urinalysis and bone marrow examinations were unremarkable.

Red discoloration in the caecum was also observed in all male dogs and one female dog in the 30 mg/kg group. Red discoloration in other parts of the intestinal tract was occasionally observed but did not appear to be treatment related. No treatment-related macroscopic abnormalities were observed after the recovery period. Organ weight analysis showed no treatment-related changes.

The most notable histopathological finding at the terminal necropsy showed bilateral degeneration of spermatozoa in the epididymides at 30 mg/kg group, and multifocal degeneration of spermatids in the seminiferous tubules at 10 and 30 mg/kg. After the 2-week recovery period, similar degenerative changes were observed in testes and epididymides of one of the two dogs in the 30 mg/kg group.

In summary, the main toxicity findings following intraperitoneal administration of TRX-E-002-1 30 mg/kg in SBECD to beagle dogs three times per week for 4 weeks were emesis, soft and/or liquid faeces, leukocytosis with elevated neutrophil and monocyte counts, red discoloration in the caecum and degenerative changes in male reproductive organs. Some of these effects were also observed at the mid-dose of 10 mg/kg, while 1 mg/kg was a clear no-effect dose. Some of the effects at the mid-dose and high-dose were not considered adverse, and hence, the NOAEL was 10 mg/kg in males and 30 mg/kg in females. No differences between sexes were apparent in toxic effects or systemic exposure, and the NOAEL for combined sexes was 10 mg/kg. The 10 mg/kg dose was also considered the highest non-severely toxic dose (HNSTD), and mean plasma AUC_last_ for combined sexes at this dose was 2310 ng h/mL on Day 26.

Degenerative changes in the testes and epididymides of male rats and dogs dosed with TRX-E-002-1 suggest that the drug may have an adverse effect on male fertility.

### Genotoxicity

Genotoxicity testing of TRX-E-002-1 was performed in an ICH-compliant test battery (see ICH Guideline S2A), comprising an in vitro bacterial reverse mutation test, an in vitro mammalian cell mutation assay and an in vivo rodent bone marrow micronucleus assay. The assays were performed in compliance with GLP regulations.

The bacterial reverse mutation test employed Salmonella typhimurium strains TA1535, TA1537, TA98 and TA100, and Escherichia coli strain WP2uvrA in a conventional plate assay, with and without exogenous metabolic activation by a rat liver post-mitochondrial fraction (S9 mix). In both tests, all bacterial tester strains showed no significant increases in the number of revertant colonies in either the absence or presence of metabolic activation. Thus, TRX-E-002-1 does not cause reverse gene mutations in bacteria under the conditions of the study.

The in vitro mammalian cell mutation assay utilised the forward mutation system at the thymidine kinase locus of the mouse lymphoma L5178Y cells using a microtitre cloning technique. TRX-E-002-1 was tested in three experimental conditions: 3-h incubation at 37 °C with and without metabolic activation system (Aroclor-induced rat liver S9 mix), and a 24-h incubation without metabolic activation (i.e. continuous treatment). Mutant frequencies for either colony size did not show statistically significant differences compared with the control values at any concentration in the absence of metabolic activation with either short-term treatment (3 h) or continuous treatment (24 h), or in either assay in the presence of metabolic activation for 3 h. Positive controls were methyl methanesulfonate in the absence of S9 and cyclophosphamide in the presence of S9 and gave the expected increases in mutant frequency assessed as either large or small colonies. Thus, TRX-E-002-1 does not cause forward gene mutation or chromosomal changes in mammalian cells under the conditions of the study.

The bone marrow micronucleus test was the in vivo part of the ICH genotoxicity testing battery and was conducted in ARC(S) Swiss mice. Eight groups of mice (10/sex/group) were given an intraperitoneal injection of vehicle (20% w/v SBECD) or Cantrixil at 50, 200 or 400 mg/kg and were killed 24 or 48 h later. Dimethyl-1,2 benzanthracene was used as the positive control. After preparation of the bone marrow cells, the number of micronucleated polychromatic erythrocytes (PCEs) was determined in 4000 PCEs per animal, and the proportion of PCEs in the total erythrocyte population in bone marrow was calculated from the examination of at least 500 erythrocytes as an index of bone marrow toxicity. The positive control group showed a statistically significant increase in the frequency of micronucleated PCEs and a reduction in the proportion of PCEs. The frequency of micronucleated PCEs was significantly increased in female mice treated with TRX-E-002-1 at 200 mg/kg (at 24 and 48 h) or 400 mg/kg (at 24 h). Slight increases in the frequency of micronucleated PCEs in male mice at 200 and 400 mg/kg were not statistically significant. The proportion of PCEs in the total erythrocyte population was decreased in both sexes at the 200 and 400 mg/kg doses at both kill times. TRX-E-002-1 is therefore considered clastogenic in this system at the described concentrations in vivo [the equivalent 200/400 mg/kg dose in a rat is 16.2 mg/kg (600 mg/m^2^)/32.4 mg/kg (1200 mg/m^2^) in a 60-kg human]. These data may imply some level of genotoxic risk for patients receiving TRX-E-002-1 at the equivalent dose in humans.

Carcinogenicity and reproductive toxicity studies of TRX-E-002-1 have not been completed. Carcinogenicity studies are not warranted to support clinical trials or marketing of therapeutics intended to treat patients with advanced cancer.

## Discussion

The introduction of combination chemotherapy several decades ago has had little impact on ovarian cancer patient survival rates, where patients who initially respond to standard of care eventually relapse with recurrent disease presenting with chemo-resistant carcinomatosis. Research indicates that tumour recurrence is caused by the expansion of chemo-resistant cancer stem cells that survive initial chemotherapy by residing in a niche microenvironment within the tumour mass. It is this population of cells with modified pro-survival mechanisms, selected for by drug pressure of treatment that is thought to be responsible for the development of chemo-resistant recurrent disease. We describe the characterisation of TRX-E-002-1, a novel SBP molecule with significant potency against clinically relevant models of ovarian cancer. TRX-E-002-1 was identified out of a medicinal chemistry-derived library of analogues with activity against a panel of cancer cells representative of different malignancies and chemo-resistant OCSCs. TRX-E-002-1 anti-tumour activity was confirmed in vivo in a highly resistant ovarian cancer animal model in the treatment of primary disease, both as a monotherapy and in combination with cisplatin. Its utility as a maintenance therapy was also demonstrated in a recurrent model of drug-resistant ovarian cancer by delaying disease recurrence. Differential survival rates exist between patients with no gross residual disease versus optimally resected residual disease [27]. Given the peritoneal cavity is the predominant site of disease in ovarian cancer, clinical researchers have employed intraperitoneal delivery of standard-of-care cytotoxics as a way to expose hypoxic, non-proliferating cells, which are resistant to chemotherapy, to higher concentrations of the therapeutic for longer periods. Based on eight randomised clinical trials the NCI encourages the combination of IV and IP chemotherapy [28]. While the combining IV with IP regimens results in more toxicity compared with IV only, the benefits outweigh the risks with overall survival for women with advanced ovarian cancer being extended by about 1 year [29]. Women were less likely to die if they received an IP component to chemotherapy with a prolonged disease-free interval and a median survival advantage of 1 month in favour of the intraperitoneal arm compared with the IV arm [30]. We describe a complete pre-clinical study justifying the use of TRX-E-002-1 in ovarian cancer. It is being developed as an IP-administered therapeutics for abdominal cancers. The primary therapeutic indication is anticipated to be first-line therapy in combination with carboplatin for epithelial ovarian cancer. Safety pharmacology, genotoxicity and toxicology studies demonstrate that TRX-E-002-1 has an acceptable toxicity profile in rats and dogs, and is without genotoxicity and cardiotoxicity at clinically relevant concentrations.

## Next steps

TRX-E-002-1 has recently achieved IND status.

A first-in-human, ascending-dose study to determine, safety, tolerability, pharmacokinetics and MTD of IP Cantrixil will be conducted in women with refractory or recurrent ovarian cancer, fallopian tube cancer or primary peritoneal cancer. In an extension cohort of this study, patient will receive Cantrixil in combination with chemotherapy.

As part of this Phase I study, circulating tumours cells in whole blood harvested from patients pre- and post-treatment will be enumerated and assessed for clonogenic potential.

TRX-E-002-1 will be further assessed against a panel of ovarian cancer cells representative of the different ovarian cancer subtypes to generate susceptibility profile. Biomarker studies are ongoing.
